# Prediction of rupture risk in cerebral aneurysms by comparing clinical cases with fluid–structure interaction analyses

**DOI:** 10.1038/s41598-020-75362-5

**Published:** 2020-10-26

**Authors:** Kwang-Chun Cho, Hyeondong Yang, Jung-Jae Kim, Je Hoon Oh, Yong Bae Kim

**Affiliations:** 1 Department of Neurosurgery, College of Medicine, Catholic Kwandong University, International St. Mary’s Hospital, Incheon, Korea; 2 Department of Mechanical Engineering, Hanyang University, 55 Hanyangdaehak-ro, Sangnok-gu, Ansan, Gyeonggi-do 15588 Korea; 3 Department of Neurosurgery, College of Medicine, Ewha Womans University, Ewha Womans University Seoul Hospital, Seoul, Korea; 4 Department of Neurosurgery, College of Medicine, Yonsei University, Gangnam Severance Hospital, 211 Eonju-ro, Gangnam-gu, Seoul, 06273 Korea

**Keywords:** Computational biology and bioinformatics, Diseases, Health care, Neurology

## Abstract

Cerebral aneurysms should be treated on the basis of accurate rupture risk prediction. Nowadays, the rupture risk in aneurysms has been estimated using hemodynamic parameters. In this paper, we suggest a new way to predict the rupture risks in cerebral aneurysms by using fluid–structure interaction (FSI) analysis for better decision-making regarding treatment. A patient-specific model was constructed using digital subtraction angiography of 51 cerebral aneurysms. For each model, a thin-walled area (TWA) was first predicted using computational fluid dynamics (CFD), and then the highest equivalent strain in the TWA was calculated with FSI by varying wall thicknesses and mechanical properties. A critical curve was made from 16 FSI results for each patient-specific model to estimate the rupture risk. On average, the equivalent strains of the ruptured aneurysms were higher than those of the unruptured aneurysms. Furthermore, the patterns of critical curves between unruptured and ruptured aneurysms were clearly distinguishable. From the rupture risk evaluation based on the cut-off value, 24 of the 27 unruptured aneurysms and 15 of the 24 ruptured aneurysms were matched with actual-clinical setting cases. The critical curve proposed in the present study could be an effective tool for the prediction of the rupture risk of aneurysm.

## Introduction

A ruptured cerebral aneurysm is the major cause of spontaneous subarachnoid hemorrhage and has a poor prognosis^1,2^. Therefore, it is very important to diagnose its rupture risk and conduct proper treatment of unruptured cerebral aneurysms before rupture occurs^3,4^. Nevertheless, the accurate prediction of the risk of aneurysm rupture in individual patient is still difficult and challenging.

The risk of rupture has been predicted in previous studies using various clinical factors such as age, hypertension, previous subarachnoid hemorrhage, and size and anatomic area of an aneurysm^1,3,4^. Hemodynamic parameters (HPs) such as wall shear stress (WSS), oscillatory shear index (OSI), and pressure have recently gained attention because they affect the formation, growth, and rupture of a cerebral aneurysm. Computational fluid dynamics (CFD) has been performed to investigate the effects of HPs and geometrical features of aneurysms on the rupture risks of aneurysms^5–9^.

However, as CFD assumes a rigid blood vessel wall, it has a natural limitation in that the effects of the wall thickness and mechanical properties of the blood vessels are ignored in the CFD analysis^10^. Because the rupture of aneurysm is significantly influenced by the wall thickness and mechanical property of aneurysms^11,12^, the accurate prediction of rupture risk could not be obtained through only CFD analysis. Generally, the thicker and the stronger an aneurysm is, the lower its rupture risk is.

In order to overcome this critical limitation of CFD, fluid–structure interaction (FSI) analysis on an aneurysm should be considered. FSI analysis is a combination of CFD and structural finite element analysis (FEA). CFD results are transferred to FEA as boundary conditions, and vice versa. FSI analysis assumes that the blood vessel is not rigid but deformable; therefore, the effects of the wall thicknesses and mechanical properties of the aneurysms could be evaluated in a more realistic manner.

In this study, a new way for more accurate prediction of rupture risk in cerebral aneurysms was suggested by analyzing FSI results of 51 cerebral aneurysms. This new approach could be an effective tool for more accurate estimation of risks of aneurysms rupture.

## Results

### FSI analysis

**Figure 1:**
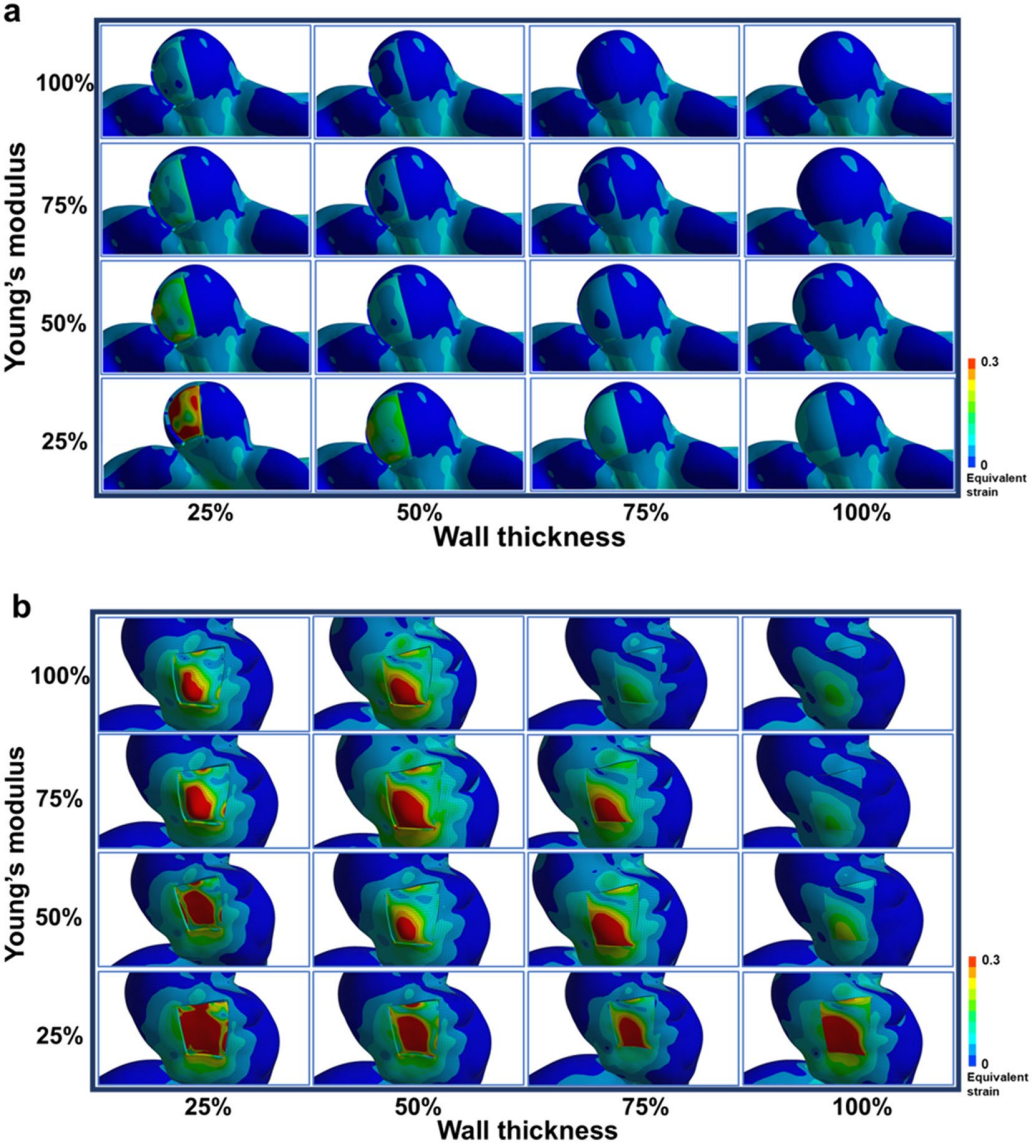
A total of 16 sets of equivalent strain contours for an unruptured aneurysm (**a**) and a ruptured aneurysm (**b**) when the wall thickness and Young’s modulus of the TWA varies from 25 to 100%. Red color in contours means that the equivalent strain in the TWA exceeds the failure strain, which indicates that rupture occurs in the TWA. TWA, thin-walled area.

Figure 1 shows the 16 FSI results of equivalent strains for the representative unruptured and ruptured aneurysms. The unruptured aneurysm was estimated to rupture only when the wall thickness and Young’s modulus became low (Fig. 1a); in contrast, in the ruptured aneurysm, a rupture was predicted to occur even with higher wall thickness and Young’s modulus (Fig. 1b). In other words, in the FSI analyses, the ruptured aneurysms could reach the rupture state faster than the unruptured ones as the wall thickness and the Young’s modulus are diminished. When both the wall thickness and the Young’s modulus were 100%, there was no difference in the equivalent strain contour between the thin-walled area (TWA) and the neighboring region. However, the difference in the contour became more obvious as the wall thickness and the Young’s modulus were reduced.

Table 1 presents a significant difference in the value of the equivalent strains for each condition of wall thickness and Young’s modulus between the unruptured and ruptured aneurysms. For all conditions, the equivalent strain of the ruptured aneurysms was significantly higher than that of the unruptured ones. In addition, the equivalent strain tended to increase with decreasing the wall thickness and the Young’s modulus.

**Table 1:** Average and standard deviation of the equivalent strains of the unruptured and ruptured aneurysms for each condition.

Conditions	Mechanical characteristics		Equivalent strain mean ± standard deviation		*P* value
	Wall thickness (%)	Young’s modulus (%)	Unruptured aneurysms (n = 27)	Ruptured aneurysms (n = 24)	
1	100	100	0.053 ± 0.026	0.094 ± 0.048	.002
2	100	75	0.071 ± 0.030	0.134 ± 0.068	< .001
3	100	50	0.105 ± 0.042	0.189 ± 0.090	< .001
4	100	25	0.206 ± 0.088	0.338 ± 0.140	.001
5	75	100	0.078 ± 0.032	0.143 ± 0.063	< .001
6	75	75	0.103 ± 0.040	0.193 ± 0.092	< .001
7	75	50	0.150 ± 0.056	0.267 ± 0.119	< .001
8	75	25	0.286 ± 0.121	0.453 ± 0.176	.001
9	50	100	0.123 ± 0.047	0.258 ± 0.167	< .001
10	50	75	0.159 ± 0.059	0.286 ± 0.122	< .001
11	50	50	0.230 ± 0.087	0.396 ± 0.164	< .001
12	50	25	0.447 ± 0.190	0.675 ± 0.281	.007
13	25	100	0.252 ± 0.103	0.455 ± 0.212	.001
14	25	75	0.337 ± 0.135	0.577 ± 0.276	.002
15	25	50	0.503 ± 0.207	0.824 ± 0.412	.007
16	25	25	1.012 ± 0.459	1.573 ± 0.823	.025

**Figure 2:**
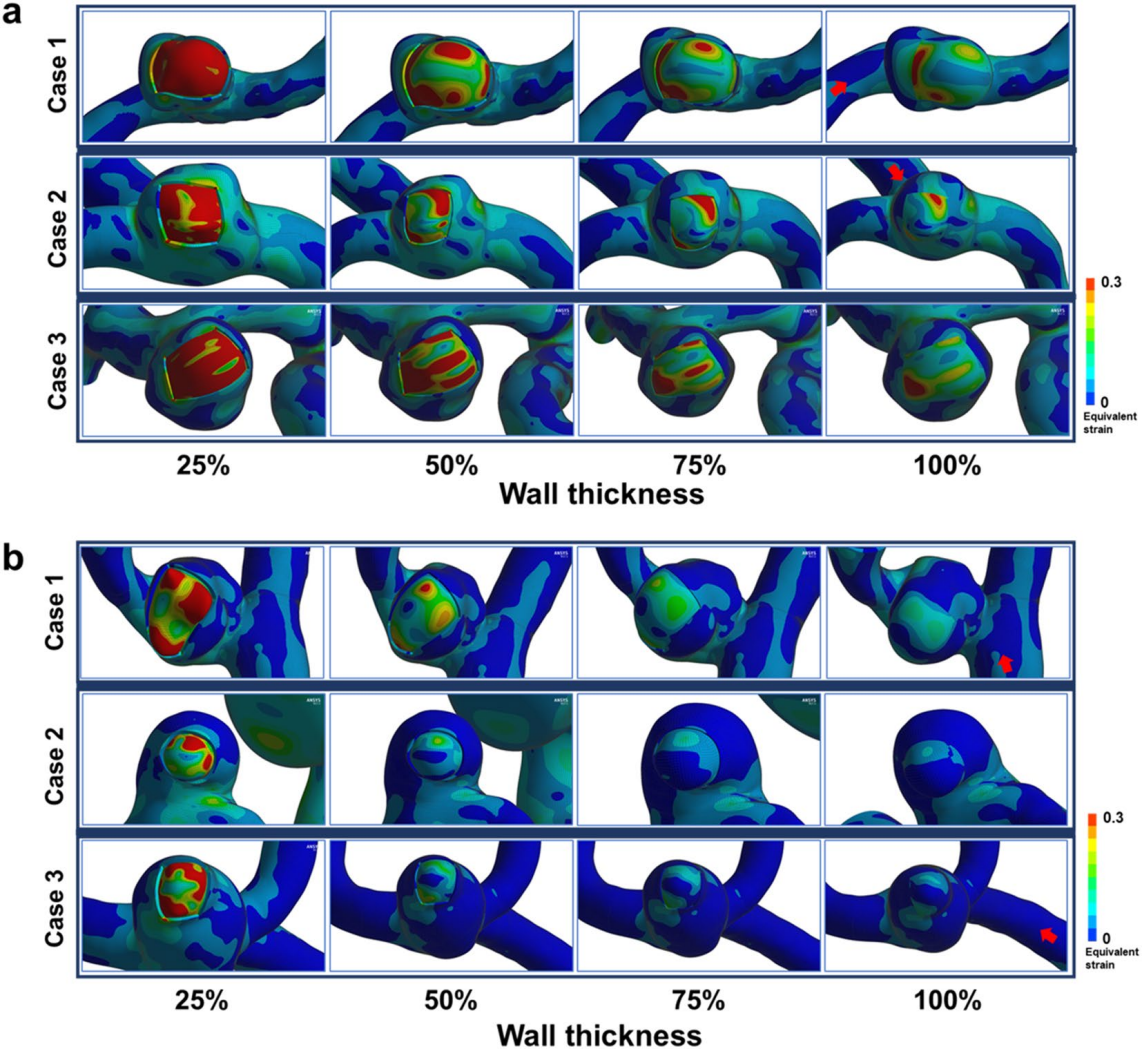
(**a**) Three typical examples of unruptured aneurysms predicted to rupture based on FSI simulation. Note that rupture was predicted to occur even with 100% wall thickness. (**b**) Three typical examples of ruptured aneurysms predicted not to rupture based on FSI simulation. In these cases, most ruptures were predicted at the smallest wall thickness. All Young’s moduli were set to 25%. Red arrows represent inflow directions. FSI, fluid–structure interaction.

However, in some cases, the prediction based on FSI analysis was not in agreement with the clinical case. Figure 2 illustrates typical examples of unmatched cases where predictions are opposite to real cases. For the cases where clinically unruptured aneurysms were predicted to rupture from the FSI analysis (Fig. 2a), higher equivalent strains were observed in the TWA even with higher wall thickness and Young’s modulus. For the opposite cases where FSI analysis predicted clinically ruptured aneurysms as unruptured ones (Fig. 2b), the equivalent strains were estimated to be low at higher wall thickness and Young’s modulus.

### Critical curve

**Figure 3:**
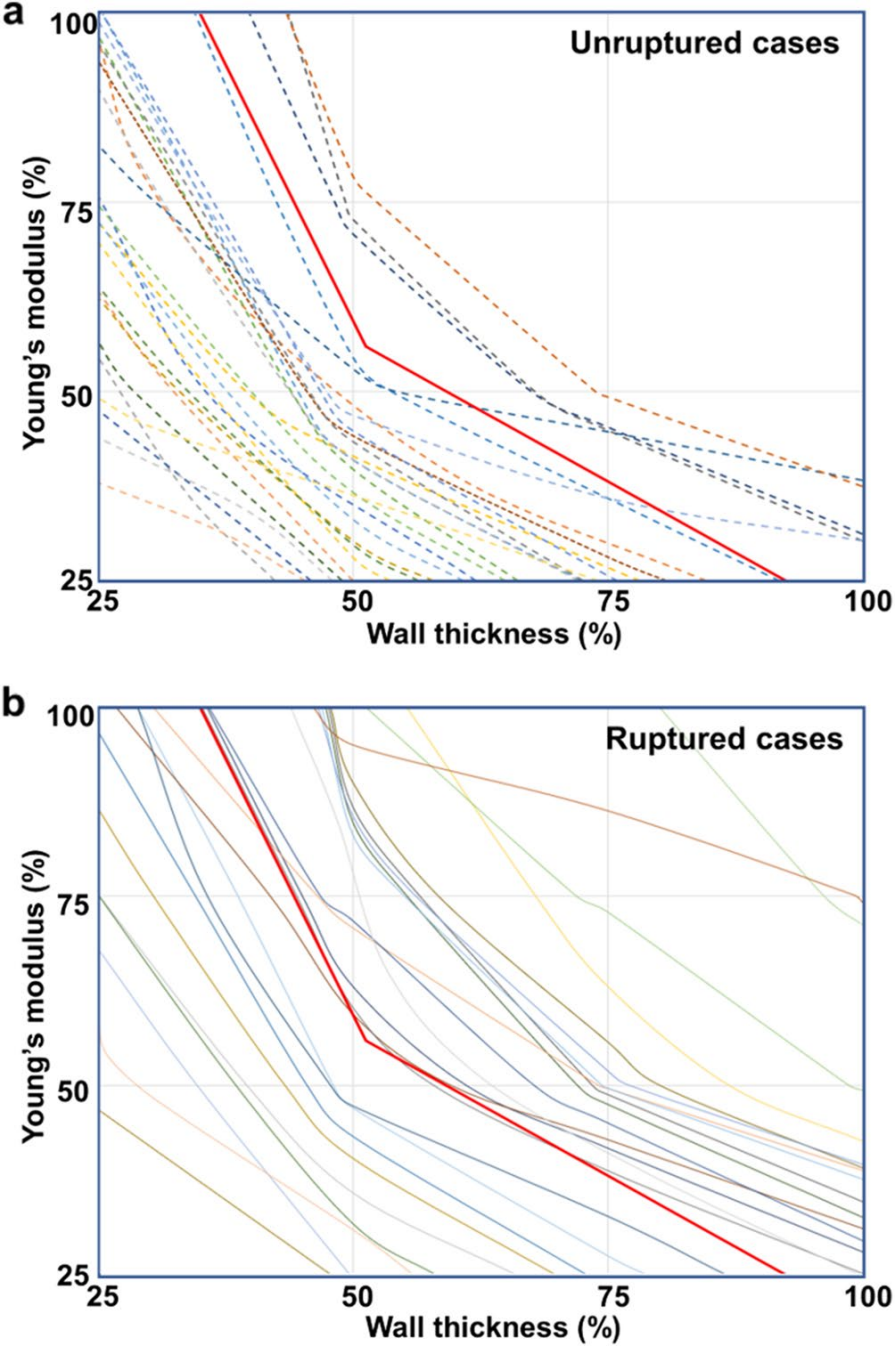
(**a**) Critical curves for 27 unruptured aneurysms. (**b**) Critical curves for 24 ruptured aneurysms. Thick red line among the curves is the reference line.

The critical curve is an intuitive graph that shows a risk of aneurysm rupture. A single curve is drawn from 16 FSI analyses for one aneurysm with the abscissa of wall thickness and the ordinate of Young’s modulus. If the higher equivalent strains in the TWA are estimated for higher wall thickness and Young’s modulus, the curve is placed at the upper right corner, which indicates that a rupture risk of corresponding aneurysm is predicted higher. On the contrary, for the critical curve located at the lower left corner, the rupture of corresponding aneurysm is predicted to occur only when the wall thickness and Young’s modulus are assumed low, and this means a lower risk of rupture. The most critical curves of unruptured aneurysms were distributed at the lower left corner (Fig. 3a), while ruptured aneurysms usually produced the critical curves at the upper right corner (Fig. 3b). However, there were some cases deviated from a general observation for both unruptured and ruptured aneurysms.

### Cut-off value

Statistical analysis was performed using Student’s *t*-test for the two cohorts of normalized area (NA) in the unruptured and ruptured aneurysms (P < 0.001). The ROC curve of the NA was made to adequately predict the possibility of rupture in cerebral aneurysms (area under the curve [AUC], 0.785). The cut-off value of the NA was measured to 0.346 (sensitivity, 62.5%; specificity, 88.9%).

## Discussion

The significance of the critical curve proposed in this study is that it could distinguish between unruptured and ruptured aneurysms, which is usually not feasible with factors currently used in the clinical setting for the prediction of their ruptures. Supplementary Information Table S1 shows that a significant difference is not observed between the two groups. Nevertheless, as listed in Table 1, when the equivalent strain values in the TWA calculated from FSI analysis are utilized, the difference between the unruptured and ruptured aneurysms becomes more obvious. This indicates that the equivalent strains from FSI analysis could play an important role in predicting the rupture risk of an aneurysm.

To improve the visibility of the cut-off value in the critical curve, the cut-off value was converted into a reference line with NA of 0.346. The critical curves above the reference line indicates the higher risks of rupture. In other words, the reference line could be regarded as the border between safe and vulnerable zone for the rupture probability. For example, three lines are above the reference line in the critical curves for the unruptured aneurysms, which indicates that the corresponding three aneurysms might be more dangerous and prone to future rupture.

The AUC value shows that the suggested method for the prediction of rupture risk is meaningful. With the cut-off value based on Youden’s index, 24 of the 27 (88.9%) unruptured aneurysms and 15 of the 24 (62.5%) ruptured aneurysms were in agreement with actual clinical results.

The three actual unruptured aneurysms, which are predicted to rupture based on the FSI analysis and cut-off value, showed the extremely reddish area in the intraoperative images; this means that they might be close to unexpected rupturing at the reddish TWAs. TWA was well observed in two cases (Supplementary Information Figures S1a and S1b), but it was not clear in another case (Supplementary Information Figure S1c). In this case, TWA might exist at the back side since the posterior wall of the aneurysm could not be fully inspected during surgery.

The case of clinically ruptured aneurysms, which are predicted not to rupture based on the FSI analysis and cut-off value, may be attributed to the inaccuracies of patient-specific models. The 3D geometric data of the ruptured aneurysms were obtained using DSA after aneurysmal sac burst, so that the geometries of aneurysms after rupture would not be exactly the same as those before rupture. Therefore, the modeling of ruptured aneurysms may not precisely reflect the original 3D geometries of corresponding aneurysms when they were intact. This is likely an important factor why the predictions for ruptured aneurysms are not good as compared with those for unruptured aneurysms. Nevertheless, the prediction of rupture risk is mainly focused to evaluate the fate of unruptured aneurysms. Therefore, we believe that the innate inaccuracies of FSI analysis in ruptured aneurysms may not greatly affect the usefulness of present methodology for prediction of rupture risk in unruptured aneurysms.

The present study had several limitations. First, the inlet and outlet conditions for the FSI analysis were not patient-specific although the modified Womersley velocity profile was applied at the inlet after adjusting the effect of inlet diameter. Furthermore, the wall thickness of the blood vessel was assumed to be uniform and 0.2 mm, but the actual wall thickness is unknown and varies among individuals. Second, the prediction of the TWA location might not be accurate. In this study, the TWA was predicted based on our previous work^13^, but it might not be perfect. The third limitation that might lower the sensitivity comes from the form of selection bias in which all cases with a ruptured aneurysm were already ruptured in time of inclusion. It seems ideal to include cases where once aneurysms are intact and FSI simulated first, and then ruptured during followed up. For the future work, we are currently constructing aneurysm DSA image database by multi center collaboration, and expect to archive longitudinal information regarding the fate of an incidentally found aneurysm.

Finally, the mechanical properties used for cerebral arteries have inherent inaccuracies^14^. As the arteries are composed of many layers^15^, they show complicated mechanical behaviors^16^. Furthermore, mechanical properties of intracranial aneurysms proof to be different from the mechanical properties of normal cerebral arteries as shown by experimental data of Scott et al.^17^. However, even though we could model all layers of arterial wall perfectly, it may not provide a meaningful additional accuracy. In addition, next to the general complicated mechanical behaviors of the arterial wall, there are also in aneurysms many disease processes which are not fully modeled present and will have an impact, such as dens atherosclerotic plaque next to the region of TWA or surrounding anatomic structure outside the aneurysmal sac. Therefore, FSI analysis on this report should be regarded as one of useful methods that may “resemble” the real hemodynamic – mechanical interaction.

In the present study, the risk of rupture in cerebral aneurysms was determined by constructing the critical curve and calculating the NA value based on the FSI analysis results. The critical curve and NA value could be helpful in determining the risk of rupture in cerebral aneurysms. Furthermore, predicting the risk of rupture in aneurysms using FSI analysis would be more realistic and accurate than using CFD alone.

What to overcome of current FSI analysis for clinical use is that it takes not inconsiderable time and trouble to simulate. However, as we are currently working on a method of automatically simulating the angiographic images from DSA to facilitate clinical application, it is expected to be resolved.

## Methods

### Data acquisition

The study protocol was approved by Yonsei University Gangnam Severance Hospital, Institutional Review Board, and the need for written informed consent was waived. Among the patients who underwent cerebral digital subtraction angiography (DSA) between April 2014 and November 2018, three-dimensional (3D) images were evaluated to determine the presence of cerebral aneurysms. Of the all patients, some received clipping or coiling based on the neurosurgeon’s decision, while others were observed their progress without treatment for aneurysms. In addition, the ruptured aneurysms were confirmed as a cause of intracranial hemorrhage when the patients had no further hemorrhage after 3 months of clinical follow-up. Patient information including sex, age, medical history, aneurysm size, aneurysm location, history of subarachnoid hemorrhage, and geographic region was recorded.

A total of 51 cerebral aneurysms (27 unruptured aneurysms and 24 ruptured aneurysms) in 51 patients were investigated in the present study. The demographic data of the patients are shown in Supplementary Information Table S1. DSA was performed on a biplane neuroangiographic unit (Artis zee biplane; Siemens Healthcare GmbH, Erlangen, Germany). After trimming and eliminating unnecessary branches, relevant images were reconstructed to patient-specific 3D models using CATIA (V5-6R2012, Dassault System, Paris) and Meshmixer (version 11.0.544, Autodesk, San Rafael, CA).

### CFD analysis

CFD was first performed with 3D model to predict a TWA on each aneurysm (Supplementary Information Figure S2a, left column), and then FSI analysis was conducted utilizing the CFD results (Supplementary Information Figure S2a, right column). The detailed procedure of TWA prediction was described in our previous work^13^. CFD was performed using ANSYS Workbench Fluent (Version 17.0, ANSYS Inc., Canonsburg, PA). Blood was assumed to be an incompressible Newtonian fluid with a density of 1,055 kg/m^3^ and a viscosity of 0.004 kg/m s; the blood vessels were assumed as a rigid wall with a non-slip condition. Pulsatile flow with a Womersley velocity profile was applied to the inlet^18^. As the outlet flow condition, the blood pressure profile was used after adjusting pressure of the carotid artery^19^.

### FSI analysis

Since FSI analysis utilizes structural FEA in addition to CFD analysis, it requires the mechanical properties and the wall thickness of the blood vessel as input conditions. Young’s modulus and Poisson’s ratio are two essential mechanical properties which are necessary to describe the elastic behavior of a blood vessel. Young’s modulus is a mechanical property that describes the ability to withstand deformations in objects undergoing forces. For example, the amount of deformation of a blood vessel with lower Young’s modulus is larger than that of a blood vessel with higher Young’s modulus under the same external force. Poisson’s ratio is a dimensionless quantity, the ratio of transverse contraction strain to longitudinal elongation strain under a longitudinal force.

The mechanical properties of cerebral aneurysms were based on experimental data from a previous study by Cebral et al. The Young’s modulus of a cerebral aneurysm has values between approximately 0.5–5.7 MPa^20^. In this study, the median of these values was used. Young’s modulus, Poisson’s ratio, and density of the cerebral artery were assumed to be 2.6 MPa, 0.49, and 1000 kg/m^3^, respectively. Cerebral arteries were assumed to fail when the strain reaches to 0.3.

The wall thickness of the cerebral arteries was determined by referencing related research works. MacDonald et al.^21^ found that the wall thickness of the cerebral arteries was widely ranged from 0.016 to 0.212 mm by using polarized light microscopy, and Valencia and Solis^22^ defined the wall thickness of the cerebral arteries as 0.2 mm. Based on these previous results, the normal wall thickness of the cerebral arteries was assumed to be 0.2 mm in this study.

The rupture of aneurysm usually occurs at a weak point where the wall thickness and the Young’s modulus are much reduced from its normal values. However, examining the precise amount of reduction in the wall thickness and Young’s modulus at the weak point is not feasible, so an indirect way was suggested to evaluate the rupture risk of the given aneurysm: the wall thickness and the Young’s modulus in the selected TWA are reduced in four steps (100%, 75%, 50%, and 25%) from their normal values, and FSI analysis is conducted for each reduced wall thickness and Young’s modulus, leading to a total of 16 FSI analyses for each aneurysm. The main idea is that there would be a different tendency in the strain results of 16 FSI analyses between unruptured and ruptured aneurysms. It should be noted that a higher strain value in the TWA under the same condition means a higher rupture risk of the aneurysm.

As boundary conditions, the inlet was all fixed, and the expansion and contraction was only allowed for the outlet. The blood flow conditions were identical to those in the CFD analysis.

### Critical curve

The critical curve was newly devised to investigate the tendency of the highest equivalent strains in the TWA calculated from 16 FSI analyses on each unruptured and ruptured aneurysm. Regardless of rupture state, a single aneurysm produces one critical curve from 16 FSI analyses with respect to the reduced wall thickness and Young’s modulus. The critical curve is the line of intersection between the surface constructed with 16 highest equivalent strain points and the plane of a failure strain of 0.3. Polynomial interpolation was performed with the 16 equivalent strain points to construct the smooth surface of equivalent strain. Supplementary Information Figure S2b shows the detailed process of how to construct the critical curve. It should be also noted that the critical curve moves further to the upper right corner as the equivalent strains in the TWA become higher under the same condition. This means that ruptured aneurysms are more likely to have critical curves located near the upper right corner because they are more likely to produce higher equivalent strains under the same wall thickness and Young’s modulus than unruptured ones.

### Statistical analysis

A NA was calculated to determine the criterion of the risk of rupture and was defined as the area between the lower part of the critical curve and the coordinate axes divided by the entire area:

$$ NA = \frac{Area \, under \, the \, curve}{{Entire \, area}} $$

The value of NA is increased as the critical curve is located at the upper right corner, but it is decreased as the critical curve moves to the lower left corner. NA has a value between 0 and 1, which means that the risk of rupture becomes higher as NA approaches to 1.

Based on the NA of each cerebral aneurysm, the cut-off value was calculated using the receiver-operating characteristic (ROC) curve with Youden’s index. All statistical analyses were performed using IBM SPSS Statistics (version 23.0, IBM Analytics).

### Ethical approval

All procedures performed in this study involving human participants were in accordance with the ethical standards of the institutional and/or national research committee and with the 1964 Helsinki declaration and its later amendments or comparable ethical standards.

### Informed consent

Name of committee that waived the informed consent for the study protocol is Yonsei University Gangnam Severance Hospital, Institutional Review Board.

## Supplementary information

Supplementary Information
