# Supplementary material for: Prediction of rupture risk in cerebral aneurysms by comparing clinical cases with fluid–structure interaction analyses
Source: Sci Rep. 2020 Oct 26;10:18237. doi: 10.1038/s41598-020-75362-5 (PMC7588470; doi:10.1038/s41598-020-75362-5)
Supplement: Supplementary file 1 — Supplementary Information [file 41598_2020_75362_MOESM1_ESM.pdf]

Supplementary Information

Prediction of rupture risk in cerebral aneurysms by comparing clinical cases with fluid-structure interaction analyses

Kwang-Chun Cho, MD[1], Hyeondong Yang, BE[2], Jung-Jae Kim, MD[3], Je Hoon Oh, PhD[2], and Yong Bae Kim, MD, PhD[4]

[1]Department of Neurosurgery, College of Medicine, Catholic Kwandong University, International St. Mary's Hospital, Incheon, Korea

[2]Department of Mechanical Engineering, Hanyang University, Ansan, Gyeonggi-do, Korea

[3]Department of Neurosurgery, College of Medicine, Ewha Womans University, Ewha Womans University Seoul Hospital, Seoul, Korea

[4]Department of Neurosurgery, College of Medicine, Yonsei University, Gangnam Severance Hospital, Seoul, Korea

KC Cho and HD Yang contributed equally to this work as co-first authors.

JH Oh and YB Kim contributed equally to this work as co-corresponding authors.

Supplementary Table S1. Patient demographics

Parameters	**Unruptured (n = 27)**	**Ruptured (n = 24)**	**P-value**
Sex			.986
Male	9 (33.3%)	9 (37.5%)	
Female	18 (66.7%)	15 (62.5%)	
Age (year)	58.1 ± 12.3	54.4 ± 12.7	.287
Location			.154
MCA	12 (44.4%)	13 (54.2%)	
Posterior circulation	0 (0.0%)	0 (0.0%)	
ICA	13 (48.1%)	6 (25.0%)	
ACA	2 (7.4%)	5 (20.8%)	
Multiplicity			.731
Yes	18 (66.7%)	18 (75.0%)	
No	9 (33.3%)	6 (25.0%)	
Neck diameter (mm)	3.6 ± 1.6	3.3 ± 0.8	.334
Maximum diameter (mm)	5.1 ± 1.9	5.7 ± 1.7	.268
Atherosclerosis			.563
Yes	21 (77.8%)	19 (79.2%)	
No	6 (22.2%)	5 (20.8%)	
Family history of hemorrhagic stroke			.326
Yes	25 (92.6%)	19 (79.2%)	
No	2 (7.4%)	5 (20.8%)	
DM			.953
No	27 (100.0%)	23 (95.8%)	
Yes	0 (0.0%)	1 (4.2%)	
HTN			.725
No	17 (63.0%)	13 (54.2%)	
Yes	10 (37.0%)	11 (45.8%)	
Ischemic stroke			1.000
No	25 (92.6%)	23 (95.8%)	

Parameters	**Unruptured (n = 27)**	**Ruptured (n = 24)**	**P-value**
Yes	2 (7.4%)	1 (4.2%)	
Lipidemia			.524
No	25 (92.6%)	24 (100.0%)	
Yes	2 (7.4%)	0 (0.0%)	
Smoking history			.372
No	20 (74.1%)	14 (58.3%)	
Yes	7 (25.9%)	10 (41.7%)	

ACA, anterior cerebral artery; DM, diabetes mellitus; HTN, hypertension; ICA, internal cerebral artery; MCA, middle cerebral artery

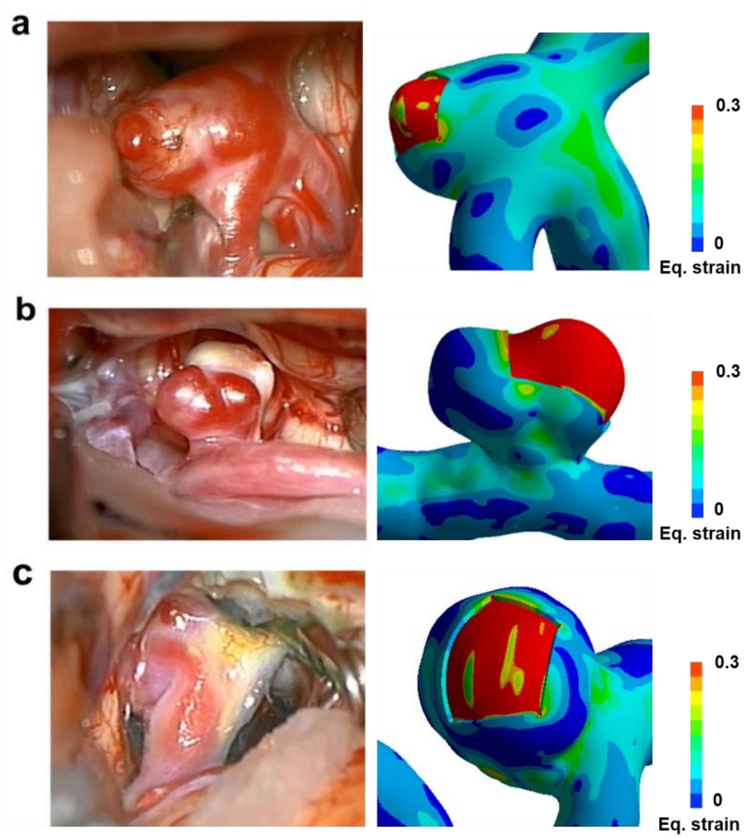

Supplementary Figure S1. Intraoperative images and FSI results of the unruptured aneurysms predicted to rupture based on the FSI analysis. The FSI results are the contour of equivalent strain when Young's modulus and wall thickness are 25 % each. TWA is well observed in two cases **(a and b)**, but in another case TWA is not clear **(c)**. FSI, fluid-structure interaction; TWA, thin-walled area

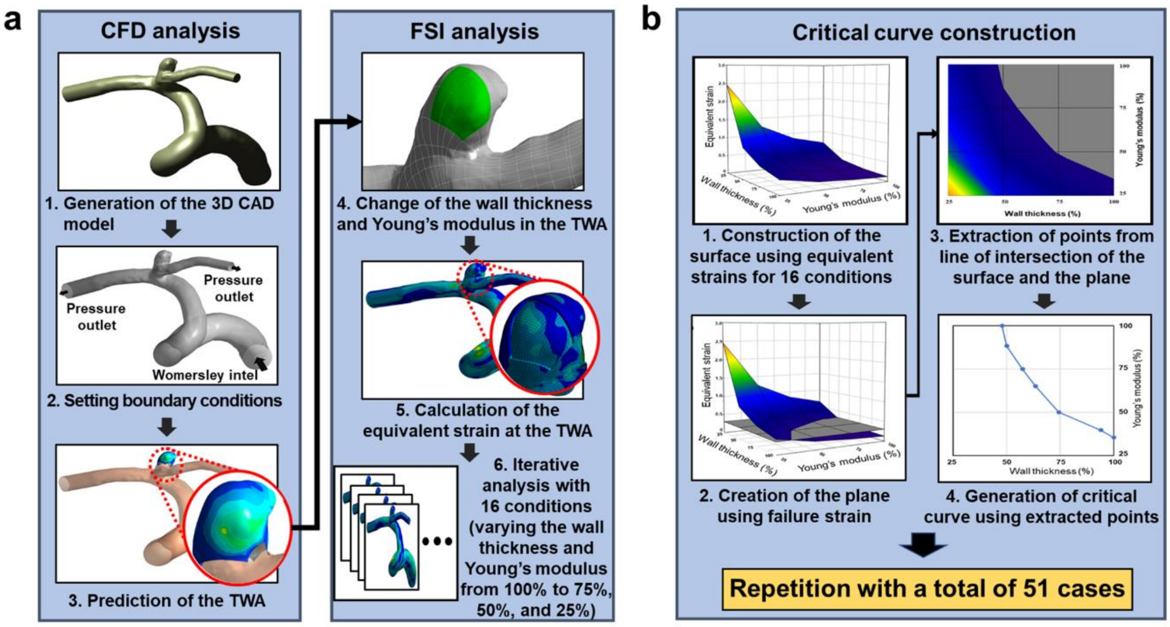

Supplementary Figure S2. (a) Entire procedure for both CFD and FSI simulations. **(b)** Procedure for critical curve construction. CFD, computational fluid dynamics; FSI, fluid structure interaction; TWA, thin-walled area; 3D, three-dimension
